# The dynamic change of neutrophil to lymphocyte ratio can predict clinical outcome in stage I-III colon cancer

**DOI:** 10.1038/s41598-018-27896-y

**Published:** 2018-06-21

**Authors:** Zhigui Li, Rui Zhao, Yaping Cui, Yong Zhou, Xiaoting Wu

**Affiliations:** Department of Gastrointestinal Surgery, West China Hospital, Sichuan University, Chengdu, Sichuan 610041 China

## Abstract

Whether the dynamic change of neutrophil to lymphocyte ratio (delta-NLR) can predict the outcome in various malignancies remained controversial. The delta-NLR has not been evaluated in colon cancer. Thus, we conducted the study to evaluate the predictive value of the delta-NLR in patients with colon cancer who underwent curative resection. Three-hundred and fifty-four patients with stage I-III colon cancer were retrospectively analysed. Clinicopathological features, preoperative NLR and postoperative NLR were collected. Prognostic factors were evaluated by univariate and multivariate analysis. The one, three and five-year overall survival rate in the delta-NLR < 0 group was 98.2%, 90.7% and 83.6%, respectively; and in the delta-NLR ≥ 0 group was 98.4%, 96.9% and 95.3%, respectively (log-rank test, *P* = 0.002). Univariate and multivariate analysis showed that there was a strong relationship between delta-NLR and overall survival. In conclusion, the delta-NLR was an independent prognostic factor for overall survival in early stage colon cancer. Patients with increased delta-NLR had an favourable clinical outcome.

## Introduction

Colon cancer remained one of the most common cancer and the leading cause of cancer-related mortality worldwide, with an increased incidence^1–3^. Previously, the prognosis of patients with colon cancer mainly relied on clinical stage at the time of diagnosis, but which was not entirely reliable. After curative resection which was the only way to cure the disease, approximately 50% of colon cancer patients developed metastatic disease. Even though histological and immunological biomarkers have been widely identified as prognostic factors, these biomarkers were usually inconvenient, time consuming and expensive to measure. Thus, there was no promising predictor of clinical outcome in colon cancer. At present, an active area of study focused on how to distinguish the subset of patients who were at high risk of recurrence and metastasis. It was necessary to explore the predictor of clinical outcome after curative resection.

Recently, the relationship between inflammation and cancer have attracted more and more attention. An increasing body of evidences showed that there was a significant relationship between systemic inflammation and relatively poor survival in various malignancies^4–7^. Systemic inflammatory responses are thought to take part in tumor progression through inducing angiogenesis and repairing DNA damage, tumor proliferation and metastasis, inhibition of apoptosis^8–11^, which also caused the changes of haematological components, like neutrophils, lymphocytes, monocytes. Neutrophils, the major components of leukocytes in blood circulation, were considered to be the first line of defense during infections and inflammation. Several studies have shown that neutrophil to lymphocyte ratio (NLR) was an independent factor for progression-free survival (PFS) and overall survival (OS) in various malignancies, including prostate cancer^12^, breast cancer^13^, colorectal cancer^14^, pancreatic cancer^15^. However, previous studies mainly focused on preoperative NLR (pre-NLR) or postoperative NLR (post-NLR). The dynamic change of preoperative and postoperative NLR (delta-NLR) has been rarely studied in colon cancer.

The aim of this study was to evaluate the predictive value of delta-NLR in patients with stage I-III colon cancer who underwent curative resection.

## Results

**Figure 1:**
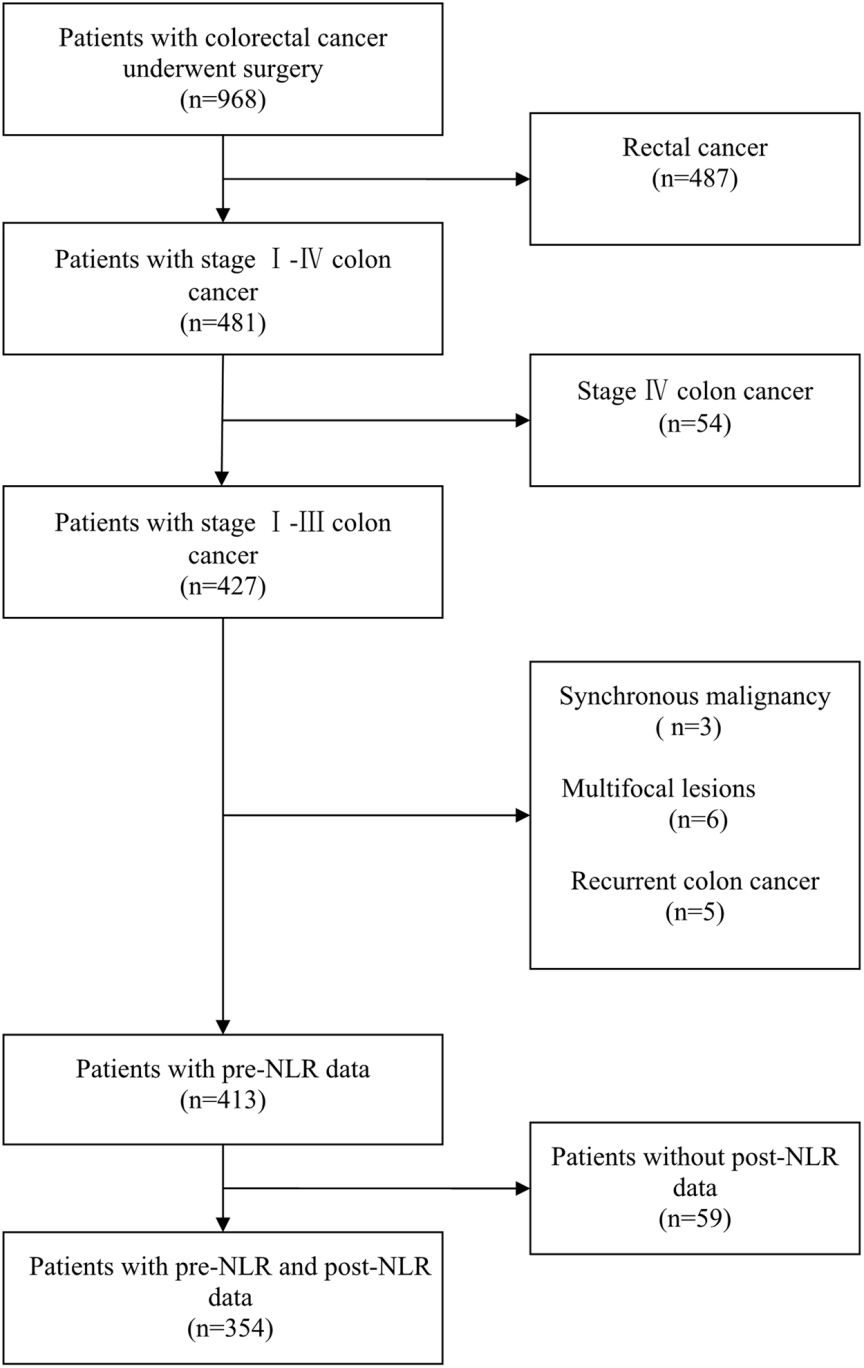
Diagram showing the process of patients selection.

In the present study, a cohort of 968 patients with colorectal cancer were screened. Among them, a total of 616 patients were excluded because of our exclusion criteria. The process of patients selection was shown in Fig. 1. In the end, three-hundred and fifty-four patients with stage I-III colon cancer and complete data were included in this study. Of these, there were 212 males, and 142 females. Age ranged from 18 to 94, with a median age of 62 years. Details about patient’s information were shown in Table 1. The median value of pre-NLR was 2.67 (1.92–3.73) and post-NLR was 2.02 (1.51–2.87). The median (range) follow-up from surgery was 49 (4–69) months. During the follow-up period, 43 (12.1%) were dead at the last follow-up time. The one, three and five-year OS rate of 354 patients was 98.3%, 92.9% and 87.9%, respectively.

**Table 1:** Baseline characteristics of patients with colon cancer.

Variable	delta-NLR < 0 (n = 226)	delta-NLR ≥ 0 (n = 128)	*P*
Age(years)			0.392
<60	106	54	
≥60	120	74	
Gender			0.001
Male	150	62	
Female	76	66	
CEA (U/ml)	2.93(1.63–9.69)	3.28(1.63–8.11)	0.993
CA199 (U/ml)	14.97(8.18–30.08)	13.63(9.64–31.85)	0.982
CA125 (U/ml)	13.79(10.41–27.55)	12.60(8.67–19.24)	0.016
pre-NLR	3.05(2.36–4.54)	1.94(1.37–2.5)	0.000
post-NLR	1.79(1.38–2.35)	2.79(2.07–3.81)	0.000
Tumor location			0.085
Right colon	148	72	
Left colon	78	56	
Tumor invasion depth			0.000
T1 + T2	14	30	
T3 + T4	212	98	
Lymph node involvement			0.058
No	160	78	
Yes	66	50	
Tumor stage			0.058
I + II	160	78	
III	66	50	
Tumor grade			0.692
Low	86	46	
Middle, high	140	82	
Hypertension			0.016
No	174	112	
Yes	52	16	
Diabetes mellitus			0.091
No	204	122	
Yes	22	6	

CEA was not indicated for 12 cases;

CA-199 was not indicated for 108 cases;

CA-125 was not indicated for 118 cases.

**Figure 2:**
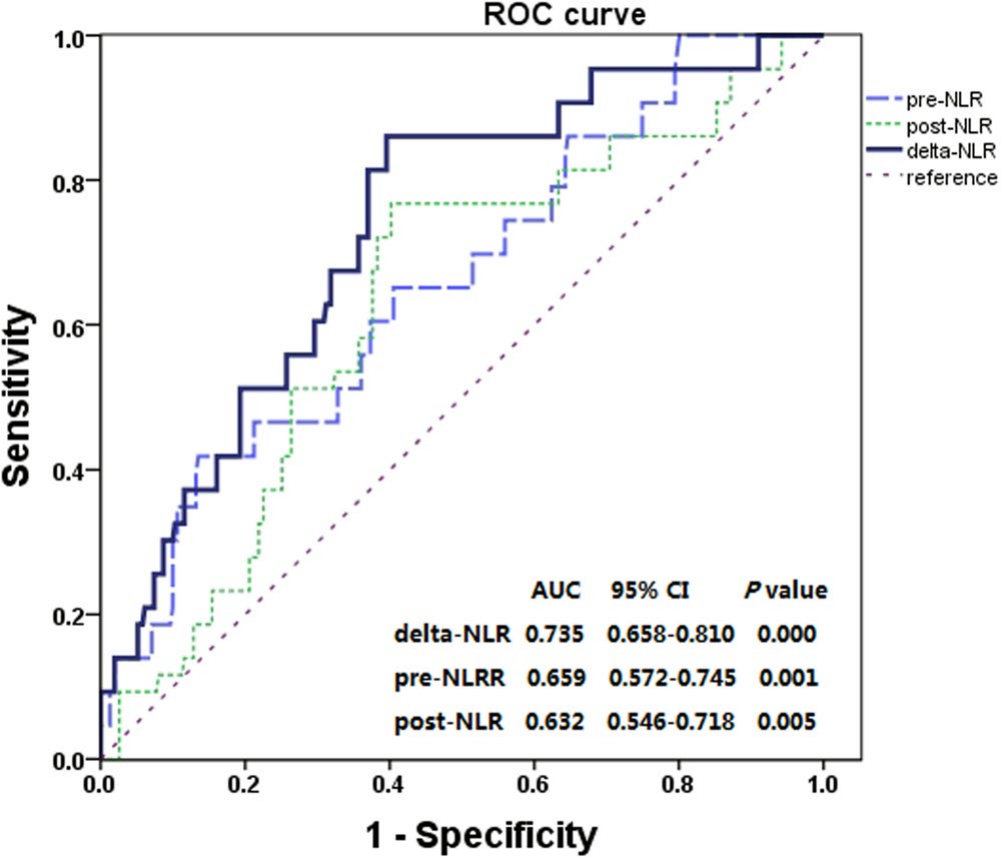
Receiver operating characteristic (ROC) curve analysis of the pre-NLR, post-NLR and delta-NLR.

In order to evaluate clinical utility of pre-NLR, post-NLR and delta-NLR, receiver operating characteristic (ROC) curves were generated. The area under the ROC curve (AUC) for OS in pre-NLR, post-NLR and delta-NLR was 0.659 (*P* = 0.001, 0.632 (*P* = 0.005) and 0.735 (*P* = 0.000), respectively. The delta-NLR tended to have greater AUC values than pre-NLR and post-NLR (Fig. 2).

**Figure 3:**
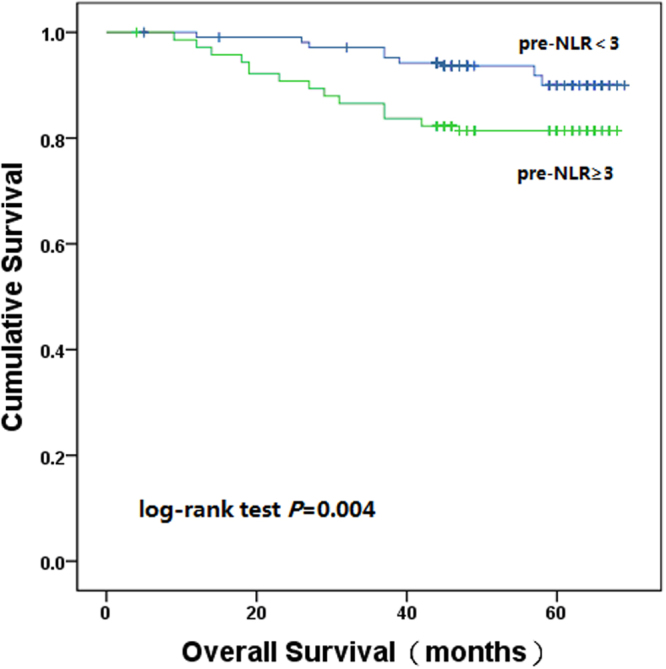
Kaplan–Meier estimates of overall survival based on the pre-NLR.

We analysed the pre-NLR with the cut-off level of 3^16^. The pre-NLR was decreased in 212 patients (59.9%), and increased in 142 patients (40.1%). When all patients were stratified into two groups by the pre-NLR; the one, three and five-year OS rate in the pre-NLR < 3 group was 99.1%, 97.2% and 92.0%, respectively; and in the pre-NLR ≥ 3 group was 97.2%, 86.6% and 81.7%, respectively (log-rank test, *P* = 0.004). During the follow-up period, 17 (8.0%) were dead in the pre-NLR < 3 group and 26 (18.3%) were dead in the pre-NLR ≥ 3 group (Fig. 3).

**Figure 4:**
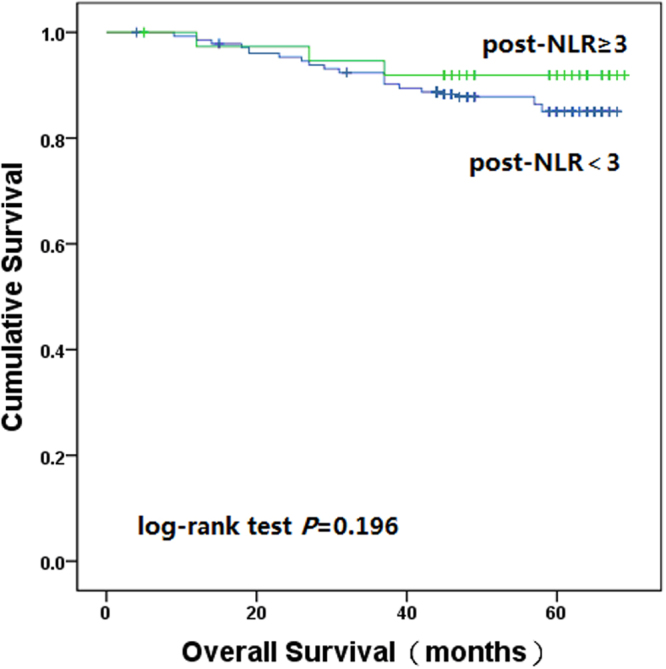
Kaplan–Meier estimates of overall survival based on the post-NLR.

We also analysed the post-NLR with the cut-off level of 3. The post-NLR was decreased in 278 patients (78.5%), and increased in 76 patients (21.5%). When all patients were stratified into two groups by the post-NLR; the one, three and five-year OS rate in the post-NLR < 3 group was 98.6%, 92.4% and 86.7%, respectively; and in the post-NLR ≥ 3 group was 97.4%, 94.7% and 92.1%, respectively (log-rank test, *P* = 0.196). During the follow-up period, 37 (13.3%) were dead in the post-NLR < 3 group and 6 (7.9%) were dead in the post-NLR ≥ 3 group (Fig. 4).

**Figure 5:**
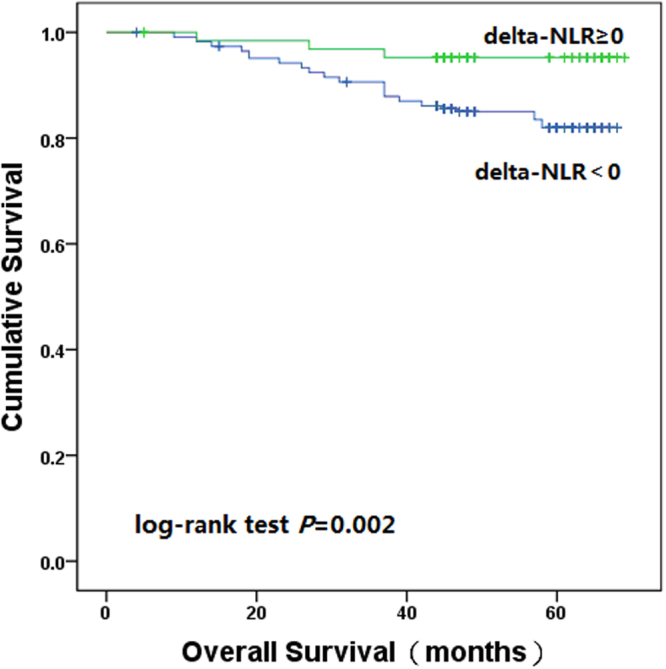
Kaplan–Meier estimates of overall survival based on the delta-NLR.

We analysed the delta-NLR with the cut-off level of 0^17^. The delta-NLR was decreased in 226 patients (63.8%), and increased in 128 patients (36.2%). When all patients were stratified into two groups by the delta-NLR, the one, three and five-year OS rate in the delta-NLR < 0 group was 98.2%, 90.7% and 83.6%, respectively; and in the delta-NLR ≥ 0 group was 98.4%, 96.9% and 95.3%, respectively (log-rank test, *P* = 0.002). During the follow-up period, 37 (16.4%) were dead in the delta-NLR < 0 group and 6 (4.7%) were dead in the delta-NLR ≥ 0 group (Fig. 5).

To evaluate the prognosis for clinical outcome in stage I-III colon cancer, potential variables were analysed in univariate and multivariate analysis. Univariate analysis showed that age, differentiation, lymph node involvement, clinical stage, pre-NLR and delta-NLR were significantly associated with OS. Because clinical stage originated from tumor depth, lymph node involvement and distant metastasis, only clinical stage entered the multivariate analysis. Multivariate analysis demonstrated that delta-NLR, pre-NLR, age, differentiation and clinical stage were independent predictors for OS (Table 2).

**Table 2:** Univariate and multivariate analysis of prognostic factors for overall survival.

Variables		Univariate analysis	Multivariate analysis (delta-NLR)		Multivariate analysis (pre-NLR)	
		*P* value	HR (95%CI)	*P* value	HR (95%CI)	*P* value
Age(years)	<60	0.012	1	0.023	1	0.013
	≥60		2.204 (1.113–4.363)		2.358 (1.200–4.631)	
Gender	Mal	0.302				
	Female					
Tumor location	Right colon	0.065	1	0.094	1	0.060
	Left colon		0.552 (0.276–1.105)		0.514 (0.257–1.028)	
Differentiation	Low	0.000	1	0.000	1	0.000
	Middle, high		0.284 (0.152–0.530)		0.324 (0.173–0.606)	
Tumor invasion depth	T1 + T2	0.114				
	T3 + T4					
Lymph node involvement	No	0.000				
	Yes					
Clinical stage	I + II	0.000	1	0.000	1	0.000
	III		5.101 (2.643–9.845)		4.771 (2.490–9.141)	
Delta-NLR	<0	0.002	1	0.001		
	≥0		0.223 (0.093–0.534)			
Pre-NLR	<3	0.004			1	0.003
	≥3				2.524 (1.356–4.698)	
Post-NLR	<3	0.196				
	≥3					

## Discussion

A century ago, Rudolf Virchow firstly found the relationship between inflammation and cancer. In recent decades, a series of studies have demonstrated that systemic inflammatory response was significantly correlated with clinical outcome in various malignancies. NLR was one of the simple and attractive biomarkers for the systemic inflammatory response. Because of low cost and easy accessiblity, NLR was widely used in clinical practice.

To the best of our knowledge, only a minority of studies evaluated the prognosis of delta-NLR^17–22^. Whether the delta-NLR was an independent prognosis in colon cancer remained unclear. The TNM classification criteria was widely used to predict the prognosis in various malignancies. Patients with stage IV colorectal cancer experienced high morbidity and impaired quality of life; the 5-year OS rate of these patients was approximately 5–15%. Thus, our study excluded stage IV colorectal cancer. A cohort study of 2536 patients conducted by Rashtak *et al*.^23^ demonstrated that NLR was no statistically significantly associated with OS and PFS in rectal cancer. For better evaluating the prognosis, we focused on stage I-III colon cancer. In the present study, we evaluated the relationship between the delta-NLR, pre-NLR, post-NLR and clinical outcome in patients with stage I-III colon cancer who underwent curative resection. The present results demonstrated that the delta-NLR was an independent prognostic factor for OS, and delta-NLR ≥ 0 was significantly associated with favourable OS.

Dan *et al*.^19^ investigated the relationship between delta-NLR and clinical outcome of small heptocellular carcinomas, and demonstrated that delta-NLR was an independent prognostic factor for OS and PFS. In their study, the survival of patients with increased delta-NLR was significantly poorer than that of decreased delta-NLR. Peng *et al*.^17^ also reported that increased delta-NLR predicted worse OS and PFS in small heptocellular carcinomas who underwent curative partial hepatectomy. Jang *et al*.^24^ reported that high post-NLR was significantly associated with unfavourable PFS and OS, but there was no relationship between delta-NLR and OS (HR 0.913; 95% CI 0.650–1.283; *P* = 0.600), PFS (HR 1.162; 95% CI 0.986–1.369; *P* = 0.074). Ohno *et al*. studied clear cell renal cell carcinoma and reported that the change of NLR (a combination of the pre-NLR and post-NLR) was an independent predictor for PFS; the 10-year PFS rate in patients with elevated post-NLR was lower than that of decreased post-NLR^20^. Min KW, *et al*.^22^ evaluated the relationship between delta-NLR and gastric cancer, and demonstrated that increased delta-NLR was associated with worse outcome. Guo D, *et al*.^21^ conducted a retrospective study to evaluate the relationship between delta-NLR and colorectal cancer, which also demonstrated that delta-NLR could predict OS, but could not predict PFS.

The decreased delta-NLR reflected increased pre-NLR and decreased post-NLR. Several recent studies demonstrated that pre-NLR was an independent prognositic factors for clinical outcome in various malignancies. Absenger G, *et al*. recruited 372 patients with stage II-III colon cancer and found that increased pre-NLR was significantly associated with worse OS and PFS^25^. Rashtak S, *et al*.^23^ recruited 1622 patients with stage I-III colon cancer and also demonstated that pre-NLR was significantly associated with worse OS and PFS. Similar to other studies, our study also confirmed that increased pre-NLR correlated with unfavourable outcome in patients with colon cancer. Several studies investigated the relationship between post-NLR and clinical outcome. Miyatani K, *et al*. reported that decreased post-NLR was significantly associated with better clinical outcome in gastric cancer^26^. Ohno Y, *et al*. reported that decreased post-NLR was a significant prognostic factor for better PFS in patients with clear cell renal cell carcinoma^20^. Our study demonstrated that increased post-NLR was slightly associated with better clinical outcome. Compared with preoperative or postoperative NLR, delta-NLR could be considered as an accurate reflection of host immune response.

Our results of delat-NLR and post-NLR were inconsistent with them. There were several reasons for this contradiction. First, Dan *et al*. and Peng *et al*. studied small heptocellular carcinomas, Jang *et al*. studied localized prostate cancer, Ohno *et al*. studied clear cell renal cell carcinoma, Min KW, *et al*. studied gastric cancer, we studied stage I-III colon cancer. It was not ruled out the possibility that the prognosis of delta-NLR in various malignancies was diverse. Second, except the study conducted by Jang *et al*.^24^, the rest of sample size was relatively small and the amount of other included patients was less than three hundred.

Until now, the underlying mechanism responsible for increased delta-NLR and long-term survival in colon cancer remained poorly understood. Several biological mechanism could contribute to the relationship. It was well known that delta-NLR was influenced by pre-NLR and post-NLR, reflecting the balance between systemic inflammatory response and immune response. An increased NLR revealed a relatively neutrophilia and lymphocytopenia. Lymphocytes inhibited the tumor proliferation and metastasis through enhancing cytotoxic cell death and cytokine production. Relative lymphocytopenia could suppress anti-tumorigenic immune response^12^. Neutrophils affected tumor by means of tumor-associated neutrophils (TANs). Similar to tumor-associated macrophages, TANs exhibited function plasticity, and could be divided into an anti-tumorigenic (N1) phenotype and a pro-tumorigenic (N2) phenotype according to their activation state, cytokine repertoire and effects on tumor growth^27,28^. N2 TANs have the ability to promote the angiogenesis and tumor proliferation through several biological mechanism. Matrix metalloproteinase-9 (MMP-9) released from N2 TANs could enhance the process of extravasation through degradation of extracellular matrix, reduce apoptosis of tumor cells and increase tumor proliferation^29,30^. Neutrophil elastase (NE), a serine protease, had the ability to promote proliferation and migration of tumor cells depending on phosphatidylinositol 3-kinase (PI-3K) signal pathway^31,32^. Cathepsin G released from N2 TANs promoted angiogenesis and migration of tumor cells through upregulating vascular endothelial growth factor (VEGF) and enhancing TGF-β signaling^33^. Other substances released from N2 TANs, such as reactive oxygen species (ROS), Arginase 1 (ARG1), also contributed to enhance tumor progression. In blood circulation, N2 TANs could induce tumor cell aggregation for helping tumor cells to survive^34^. Nevertheless, TANs could be modulated and polarized toward N1 phenotype in the presence of type-I interferon or absence of TGF-β^35,36^. In stark contrast to the pro-tumorigenic function, N1 TANs hindered tumor proliferation and metastasis via producing more superoxide and hydrogen peroxide, promoting CD8+ T cell recruitment and activation, expression of higher levels of Fas, TNF-α and ICAM-I^27,37^. It was hypothesized that TANs presented pro-tumorigenic function during the preoperative period, TANs presented anti-tumorigenic function during the postoperative period. Our study demonstrated that increased post-NLR was associated with better clinical outcome and increased pre-NLR was associated with unfavorable clinical outcome, which supported above hypothesis.

It was noted that there were several limitations in the present study. First, this was a retrospective study at a single institution, so the results may not be generalizable. Second, the weakness of small sample size and relative short follow-up period could not be neglected. Third, data of adjuvant chemotherapy were not routinely available in this analysis.

In conclusion, irrespective of the limitations mentioned above, our study showed that the delta-NLR was an independent prognostic factor for patients with early stage colon cancer who underwent curative resection, and increased delta-NLR was significantly associated with better OS. Further large-scale and well designed prospective studies are still needed to confirm the present results.

## Materials and Methods

### Study population

Patients with colon cancer in our study were recruited between February 2012 and March 2015 from the Department of gastrointestinal surgery, West China Hospital. All participants signed the informed consents. The study adhered to the principles of the Declaration of Helsinki, and was approved by the Ethics Committee of West China Hospital of Sichuan University. All patients with colon cancer underwent curative resection and were confirmed by a postoperative pathologic examination.

In the present study, the inclusion criteria were as follows: (1) age 19–94 years; (2) stage T1–4, N0–2, M0; (3) no preoperative antitumor treatment, such as radiotherapy, chemotherapy; (4) survival time beyond 3 months; (5) adequate bone marrow function (absolute neutrophil count no less than 1.5 × 10^9^/L); (6) patient with preoperative and postoperative blood routine tests.

Exclusion criteria included the following: (1) clinical history with hemopathy or synchronous malignancy; (2) with metastasis; (3) recurrent colon cancer; (4) multifocal lesions; (5) loss to follow-up within 3 months after sugery. In the light of our inclusion and exclusion criteria, a total of 354 patients with pre-NLR and post-NLR data were enrolled in this study.

### Patients characteristics

Clinical characteristics including age, gender, tumor location, medical history, carcinoembryonic antigen (CEA), carbohydrate antigen 199 (CA-199), carbohydrate antigen 125 (CA-125) and pathological features were obtained through a retrieval of our prospectively maintained database. Clinical stage was determined according to the 7th edition of the American Joint Committee on Cancer (AJCC) staging system^38^.

### Peripheral blood parameters

Preoperative blood routine test was obtained within one week before curative resection; postoperative blood routine test was taken during the recovery period of one to three months after surgery. Leukocyte, neutrophil and lymphocyte were collected from the blood routine test. The normal range of leukocyte count was from 4 × 10^9^/L to 10 × 10^9^/L. The NLR (a single value for each patient) was calculated as the absolute neutrophil count divided by the absolute lymphocyte count. The delta-NLR was defined as post-NLR minus pre-NLR^19^.

### Follow-up visit

All patients were suggested to return for a follow-up visit at the first month after surgery, every 3 months during the following 3 years, and every 6 months in the subsequent years. The follow-up period was defined as the duration of time from the date of surgery to the date of the last known contact or the date of death. OS was calculated from the date of surgery to the date of death by any causes. The last follow-up date was the end of December 2017.

### Statistical analysis

We performed the ROC curve analysis to count the AUC and then determine the optimal prognosis. Normally distributed data were presented as mean ± standard deviation and abnormally distributed data as median (quartile range). We compared categorical data by the chisquare test. The univariate analysis was performed by the Kaplan–Meier method with a log-rank test. Potential effects of clinicopathological variables on OS were firstly examined using univariate analysis. Multivariate Cox-regression analysis was used to further assess the variables which was proved significant in univariate analysis. Hazard ratio (HR) and 95% confidence interval (CI) estimated from the Cox analysis were reported. All statistical analyses were performed using SPSS 19.0 software (SPSS, Inc., Chicago, IL, USA), and two-sided *P* value of less than 0.05 was considered statistically significant.
